# Impact of Stress on Premenstrual Syndrome Among Young Women: A Cross-Sectional Study

**DOI:** 10.7759/cureus.74622

**Published:** 2024-11-27

**Authors:** Diya Trivedi, Karan Patel, Kamleshkumar G Jain

**Affiliations:** 1 Community Medicine, GCS Medical College, Hospital and Research Center, Ahmedabad, IND

**Keywords:** cross-sectional study, gujarat, premenstrual syndrome, stress, young women

## Abstract

Background and aims: Premenstrual syndrome (PMS) is a common condition affecting young women, characterized by emotional, behavioral, and physical symptoms. Stress is believed to exacerbate PMS symptoms, yet the relationship between stress and PMS remains underexplored in the Indian context, particularly among young women in urban areas like Ahmedabad. This study aims to assess the impact of stress on PMS among young women aged 18-21 years residing in Ahmedabad, Gujarat.

Materials and methods: A cross-sectional study was conducted among 473 young women aged 18-21 years in Ahmedabad, Gujarat, using a stratified random sampling technique. Data were collected using a validated questionnaire that assessed PMS symptoms and perceived stress levels. Statistical analysis was performed using chi-square tests to determine the association between stress and PMS.

Results: Participants found to have moderate to severe PMS were 167 (35.3%). The most commonly reported symptoms included irritability, fatigue, and breast tenderness. A majority (84.4%) of the participants were having moderate stress. A significant positive correlation was found between high-stress levels and the severity of PMS (p < 0.01). The amount of menstrual flow was found to be associated with PMS in this study.

Conclusions: The study highlights the significant impact of stress on the severity of PMS among young women in Ahmedabad, Gujarat. Considering higher percentages of stress levels among participants, there is a need for stress management interventions to alleviate PMS symptoms among the study participants.

## Introduction

Premenstrual syndrome (PMS) encompasses clinically significant somatic and physiological manifestations during the luteal phase of the menstrual cycle, leading to substantial distress and impairment in functional capacity [1]. These symptoms disappear within a few days of the onset of menstruation. It has been found through various epidemiological surveys that as many as 75% of women of reproductive age have been experiencing one or more symptoms that can be linked to PMS [2]. Symptoms can vary widely, from mild discomfort to severe distress, affecting daily functioning and quality of life. The reported prevalence of PMS in India ranges from 14.3% to 74.4% [3].

Various causes for PMS are believed to be cyclic hormonal changes, serotonin and epinephrine levels, lifestyle, and even stress. Stress has been identified as a potential exacerbating factor for PMS, with several studies suggesting that higher levels of stress may lead to more severe symptoms [4]. The relationship between stress and PMS, however, remains complex and multifactorial [5]. Psychological stress may influence the severity of premenstrual symptoms by activating the hypothalamic-pituitary axis, which can alter ovarian hormone levels. It may also stimulate the sympathetic nervous system, leading to imbalances in neurotransmitters and other brain processes [6]. Although the relationship between stress and PMS has been widely explored in Western populations, there is a lack of research on this aspect in the Indian context. Given the widespread taboos surrounding menstruation in a country like India, there is a pressing need for studies focused on the prevalence of PMS and the impact of stress on the severity of PMS among young women. With this background, a study was planned to assess the prevalence of PMS and perceived stress and to explore the association between them among young women residing in Ahmedabad city in India.

The objective of this study is to determine the prevalence of PMS among females aged 18-21 years, assess the level of stress among the study population using the Perceived Stress Scale (PSS), and evaluate the impact of stress on the severity of PMS among the study participants.

## Materials and methods

Study design

This cross-sectional study was conducted among 473 women aged 18-21 years residing in Ahmedabad, India. The study duration was from August 2022 to January 2023. A sample size of 417 was calculated using the formula \[ \frac{4pq}{L^2} \] where p is the prevalence of PMS (48%) and L is 10% of the prevalence (4.8) [7].

Study population and sampling

The study population comprised females aged 18 to 21 years residing in Ahmedabad City. The age group 18 to 21 years is a transitional period when young women often experience significant changes, including entering college or the workforce, living independently, or navigating new social and academic pressures. These life transitions can increase susceptibility to stress, making it an important time to study its effects on PMS. Multistage sampling was used. In the first stage, two zones of the Ahmedabad Municipal Corporation were selected randomly out of the seven available zones using simple random sampling. In the second stage, one ward was selected randomly from each zone. The Urban Health Center of a selected ward was approached to get the details of the areas. From the list of areas, simple random sampling was applied using the lottery method, and one area was selected from each ward. House-to-house visits were carried out by the investigators in a selected area for interviewing the study participants fulfilling the inclusion criteria. An equal number of participants were included from each age.

Inclusion and exclusion criteria

The inclusion criteria include females between 18 and 21 years of age who have attained menarche and are willing to give informed consent.

The exclusion criteria include participants with pre-diagnosed medical and gynecological problems like polycystic ovarian syndrome, hypothyroidism, diabetes, asthma, migraine, epilepsy, pelvic inflammatory disease, amenorrhea, and endometriosis who were excluded. Females who were using medications affecting menses (antidepressants, anticonvulsants, vitamins, hormones) within the past three months were also excluded.

Data collection

Data were collected using a structured, self-administered questionnaire. The questionnaire included sections on demographic information, menstrual history, PMS symptoms (assessed using the Premenstrual Symptoms Screening Tool (PSST)), and perceived stress levels (assessed using the PSS). Participants were inquired about any PMS-related symptoms within the past three cycles. Kuppuswami classification was used to assess the socioeconomic status of the participants [8].

PSST

PSST [9] is a self-report instrument that assesses the presence and severity of premenstrual symptoms. It was developed by Kristin Lukacs and Joan C. Chrisler in 2013. It consists of 19 items, and each item is rated on a four-point Likert scale, ranging from 0 (not at all) to 3 (very much). It is a 19-item instrument consisting of two domains:

First domain: It includes 14 premenstrual symptoms that must start before the period and stop within a few days of bleeding. It involves (1) anger/irritability, (2) anxiety/tension, (3) tearful/increased sensitivity to rejection, (4) depressed mood/hopelessness, (5) decreased interest in work activities, (6) decreased interest in home activities, (7) decreased interest in social activities, (8) difficulty in concentrating, (9) fatigue/lack of energy, (10) overeating/food cravings, (11) insomnia, (12) hypersomnia, (13) feeling overwhelmed or out of control, and (14) physical symptoms such as breast tenderness, headaches, joint/muscle pain, bloating, and weight gain.

Second domain: It includes five items that evaluate the impact of symptoms on women’s functioning as interference with (1) work efficiency or productivity, (2) relationships with co-workers, (3) relationships with family, (4) social life activities, and (5) home responsibilities.

Women having at least four symptoms from the first domain or at least one out of the first four symptoms or at least one impact from the second domain were considered as having moderate to severe PMS.

The PSST is a reliable and valid measure for assessing premenstrual symptoms in females. The internal consistency of the scale is high, with Cronbach's alpha coefficients ranging from 0.88 to 0.94. The test-retest reliability of the scale is also good, with correlation coefficients ranging from 0.70 to 0.90 [10].

PSS

PSS [11] is a self-report instrument that measures the degree to which individuals perceive their life situations as stressful. It was developed by Sheldon Cohen, Ronald C. Kessler, and Lynn Underwood Gordon in 1983. The PSS is widely used in research and clinical settings to assess stress levels in various populations. It consists of 10 items, and each item is rated on a five-point Likert scale, ranging from 0 (never) to 4 (very often). The scale is designed to measure how often the respondent has experienced feelings of stress or strain in the past month. To interpret the PSS, responses to positively phrased items (typically items 4, 5, 7, and 8) are reversed - where "0" becomes "4," "1" becomes "3," and so forth. These reversed scores, along with the scores of the remaining items, are then summed to calculate the total score. Scores range from 0 to 40, with higher scores indicating higher levels of perceived stress. Scores ranging from 0 to 13 would be considered low stress, a score of 14-26 would be considered moderate stress, while scores ranging from 27 to 40 were considered as high perceived stress.

The PSS is easy to administer and has good reliability and validity. The internal consistency of the PSS is high, with Cronbach's alpha coefficients ranging from 0.78 to 0.91. The test-retest reliability of the scale is also good, with correlation coefficients ranging from 0.55 to 0.85.

Ethical considerations

The study protocol was reviewed and approved by the Institutional Ethics Committee of GCS Medical College, Hospital and Research Center, Ahmedabad, India. Written informed consent was obtained from all participants prior to data collection.

Statistical analysis

Data were analyzed using IBM SPSS Statistics for Windows, Version 26 (Released 2019; IBM Corp., Armonk, New York, United States). Descriptive statistics were used to summarize the demographic characteristics and prevalence of PMS and stress levels. Chi-square tests were used to assess the association between stress levels and PMS severity.

## Results

Demographic characteristics

A total of 473 participants have participated in the study. The mean age of participants was 19.50 with a standard deviation of 1.2 years. Equal numbers of participants were included from each age stratum. Among them, 65.54% belonged to the upper middle class, 18.60% to the upper class, and the remaining 15.86% belonged to other classes as per the Kuppuswami scale. A total of 62.58% of participants fall under the normal category of BMI (a BMI of 18.5-24.9 is considered normal). Around 17.12% were underweight (BMI < 18.5) and 20.30% were overweight (BMI > 25).

The mean age of menarche was 13.41 years. The average length of the menstrual cycle was 27.39 days, with a range of 21 to 40 days. The average duration of menstruation was 4.87 days, with a range of 2 to 10 days. Additionally, 74.42% of participants had a duration of flow between three to five days. Approximately 40.80% of participants experience dysmenorrhea, either occasionally or consistently, during their menstrual cycle. Around 25.57% of participants have an altered duration of menstruation.

**Figure 1:**
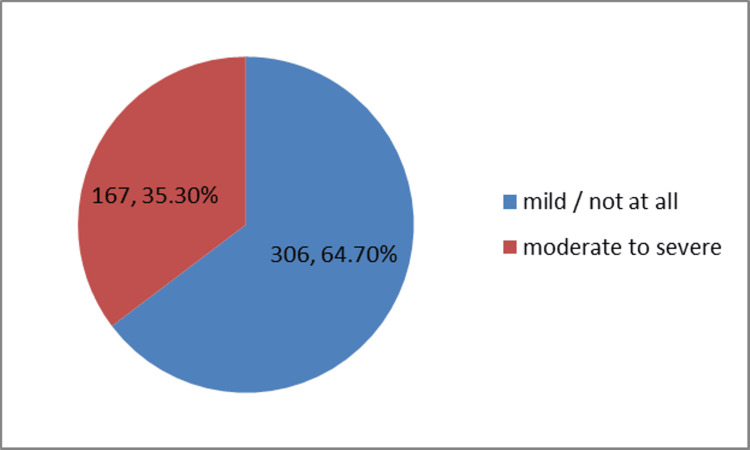
Prevalence of premenstrual syndrome among study participants (N = 473)

**Figure 2:**
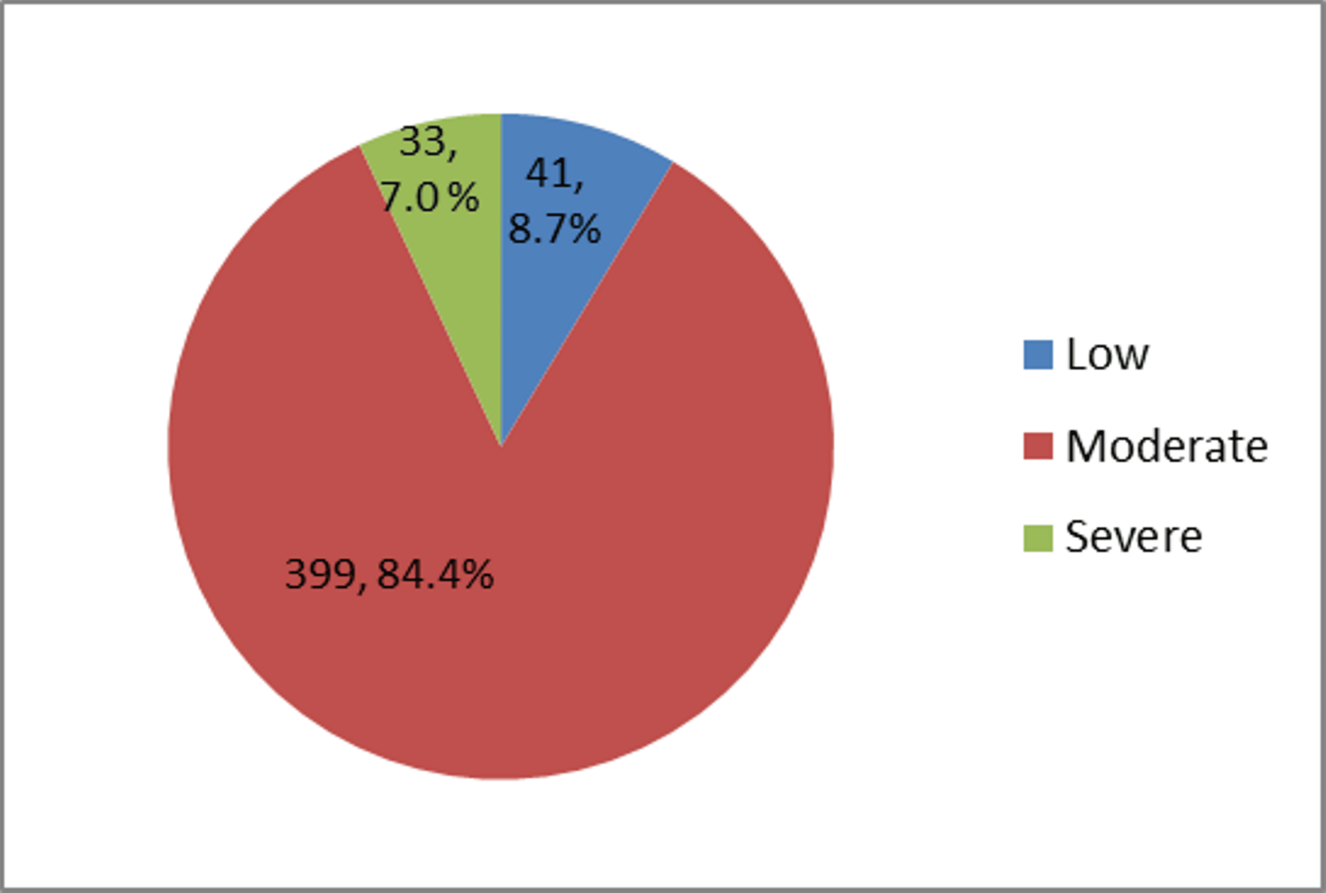
Prevalence of stress among study participants (N = 473)

Participants found to have moderate to severe PMS were 167 (35.3%). The most commonly reported symptoms included irritability, fatigue, and breast tenderness (Figure 1). The majority (84.4%) of the participants were having moderate stress as per the PSS scoring (Figure 2).

Among the participants having lower levels of stress, only 17.03% had moderate to severe PMS, whereas for those with severe stress, moderate to severe PMS was quite high (63.63%). A significant positive association was found between high stress levels and the presence of PMS symptoms (p < 0.001) (Table 1).

**Table 1:** Association between premenstrual syndrome and stress among study participants (N = 473) Note: figures in the parenthesis are column-wise percentages

PMS	Stress*			Total	Chi-square (p-value)
	Low	Moderate	Severe		17.6 (<0.001)
Present (moderate to severe)	7 (17.03%)	139 (34.84%)	21 (63.63%)	167	
Absent (mild or not at all)	34 (82.93%)	260 (65.16%)	12 (36.36%)	306	
Total	41	399	33	473	

Table 2 shows a statistically significant association between heavy menstrual flow and moderate to severe PMS (p = 0.018), suggesting that heavier flow correlates with more severe symptoms. No significant associations were found for dysmenorrhea, cycle regularity, or duration of menstruation with PMS severity.

**Table 2:** Association between premenstrual syndrome and menstrual history of the study participants (N = 473)

Variables	PMS		Total	Chi-square value	p-value
	Moderate to severe n (%)	No/mild PMS n (%)			
Dysmenorrhea					
Yes	30 (30.30%)	69 (99.69)	99 (20.93%)	3.208	0.201
No	108 (38.57%)	172 (61.43%)	280 (59.20%)		
Sometimes	29 (30.85%)	65 (69.15%)	94 (19.87%)		
Inter menstrual bleeding					
Yes	17 (54.84%)	14 (45.16%)	31 (6.55%)	5.897	0.052
No	129 (33.42%)	257 (66.58%)	386 (81.61%)		
Sometimes	21 (37.50%)	35 (62.50%)	56 (11.84%)		
Amount of flow					
Light	28 (42.42%)	38 (57.58%)	66 (13.95%)	12.748	0.018
Normal	103 (30.56%)	234 (69.44%)	337 (71.25%)		
Heavy	36 (51.42%)	34 (48.58%)	70 (14.80%)		
Duration of menstruation					
<3 days	0 (0%)	4 (100%)	4 (0.84%)	0.615	0.590
3-5 days	124 (35.23%)	228 (64.77%)	352 (74.42%)		
>5 days	43 (36.75%)	74 (63.25%)	117 (24.73%)		
Regularity of cycle					
Regular	142 (35.50%)	258 (64.50%)	400 (84.57%)	0.042	0.837
Irregular	25 (34.25%)	48 (65.75%)	73 (15.43%)		

## Discussion

The current study has evaluated the prevalence of PMS and stress among young women residing in Ahmedabad city in India. In this study, 35.3% of participants reported experiencing moderate to severe PMS. The prevalence of PMS observed in this study is comparable to that reported in other studies. For instance, a study by Bansal et al. (2017) [12] conducted among college students in North India found a PMS prevalence rate of 35.9%, with a similar percentage of participants reporting severe symptoms. In a study conducted by Upadhyay et al. (2023) [13], the prevalence of PMS was 86%, which was higher as compared to the current study. Internationally, a study by Halbreich et al. (2003) [14] found that the prevalence of moderate to severe PMS among women in the USA was around 30%. These findings highlight the substantial burden of PMS among young women across different cultural contexts, emphasizing its global relevance.

The current study noted a significant positive association between high-stress levels and the presence of PMS symptoms (p < 0.001), with the majority of participants (84.4%) experiencing moderate stress and 7% reporting high-stress levels. These results are consistent with those of a study by Kamat et al. (2020) [15], which identified stress as a significant predictor of PMS among female college students in India. Similarly, a study conducted by Alshdaifat et al. (2022) [16] in Jordan reported that higher stress levels were associated with more severe PMS symptoms, underscoring the universal nature of this relationship. Abu et al. (2021) stated that all PMS symptoms were significantly associated with depression, anxiety, and stress among the participants [17]. The role of academic pressure as a primary stressor, as reported by the participants in their study. The current study also reported similar results. These findings further underscore the need for targeted interventions to manage stress in this population.

In studying menstrual characteristics, a significant association was observed between PMS and the amount of menstrual flow (p = 0.018), with a higher prevalence of PMS among participants with heavy menstrual flow. This finding aligns with the results of a study by Durairaj and Ramamurthi (2019) [18] in India, which reported a higher incidence of PMS among women with heavy menstrual bleeding. However, no significant association was found between PMS and other menstrual characteristics such as dysmenorrhea, intermenstrual bleeding, or the regularity of the menstrual cycle in the present study.

The findings of this study suggest that interventions aimed at reducing stress could be beneficial in alleviating PMS symptoms among young women. Educational programs could incorporate stress management techniques as part of their health promotion activities. Further, longitudinal studies are needed to explore the causal relationship between stress and PMS and to identify other potential risk factors for severe PMS in this population.

Strengths and limitations

A major strength of this study is the large sample size and the use of validated tools for assessing PMS and stress. However, the cross-sectional design limits the ability to establish a causal relationship between stress and PMS. Additionally, self-reported data may be subject to recall bias.

## Conclusions

Among female participants aged 18 to 21 years, the prevalence of moderate to severe PMS was around 35.5%. Around 84% of the participants had moderate levels of stress as per the PSS. A significant association was found between the level of stress and PMS. The findings of this study highlight the significant burden of PMS among young women and its association with stress and certain menstrual characteristics. The findings underscore the need for targeted interventions to reduce stress and improve the quality of life for young women affected by PMS. Future research should focus on exploring the underlying mechanisms linking stress and PMS and developing targeted strategies to manage these conditions.
